# Outcomes of Different In Vitro Maturation Procedures for Oocyte Cryopreservation for Fertility Preservation and yet Another Live Birth in a Cancer Patient

**DOI:** 10.3390/life13061355

**Published:** 2023-06-09

**Authors:** Daniela Nogueira, Carole Fajau-Prevot, Muriel Clouet, Patrick Assouline, Marion Deslandres, Marie Montagut

**Affiliations:** 1 Fertility Institute La Croix du Sud—INOVIE Fertilité, Clinique La Croix du Sud, 52 Chemin de Ribaute, 31130 Toulouse, France; marie.montagut@inovie.fr; 2 ART Fertility Clinics, Abu Dhabi P.O. Box 60202, United Arab Emirates; 3 Department of Gynecology, Clinique La Croix du Sud, 54 Chemin de Ribaute, 31130 Toulouse, France; drfajauprevot@orange.fr (C.F.-P.); dr.m.clouet@orange.fr (M.C.); 4 Department of Obstetrics and Gynecology, Clinique La Croix du Sud, 52 Chemin de Ribaute, 31130 Toulouse, France; patrickassouline@hotmail.com; 5 Department of Gynecology, Institut Universitaire du Cancer de Toulouse, 1 Av. Irène Joliot-Curie, 31100 Toulouse, France; deslandres-cruchant.marion@iuct-oncopole.fr

**Keywords:** IVM, OPU-IVM, OTO-IVM, ex vivo oocyte extraction, immature oocyte, fertility preservation, ovarian tissue, cancer patients, oocyte cryopreservation, vitrification

## Abstract

To ensure patient care in an oncological fertility preservation (FP) programme, specialists must provide technology that best suits the patients’ clinical conditions. In vitro oocyte maturation (IVM) and ovarian tissue cryopreservation (OTC) are possible fertility preservation treatments for women in need of urgent oncological treatment. IVM consists of the retrieval of immature oocytes from small antral follicles, with no or minimal ovarian stimulation by gonadotropins. Therefore, IVM has become a pertinent option for fertility preservation, especially for cases whereby ovarian stimulation is unfeasible or contra-indicated. Existing data on immature oocytes, retrieved transvaginally (OPU-IVM) or extracted from ovarian tissue ‘ex vivo’ (OTO-IVM), are still limited on technical consistency, efficacy, and safety. The present retrospective cohort study includes 89 women undergoing fertility preservation using IVM methodologies and 26 women undergoing ovarian stimulation (OS) in concomitant period. In total, 533 immature oocytes were collected from IVM patients, achieving a maturation rate of 57% and 70% in OTO-IVM and 73% and 82% in OPU-IVM at 24 h and 48 h in culture, respectively. The observed high maturation rates might be due to the use of patients’ serum in its innate status, i.e., without heat-inactivation. This permitted 7.6 ± 5.7 and 4.6 ± 4.9 oocytes to be vitrified in OTO-IVM and OPU-IVM, respectively, compared to 6.8 ± 4.6 from OS patients. Regarding OS patients, two of them underwent embryo transfer following the insemination of warmed oocytes after complete remission, resulting in a single live birth from one patient. Upon follow-up of two OTO-IVM patients after the termination of their oncological treatment, a total of 11 warmed oocytes lead to a transfer of a single embryo, but pregnancy was not achieved. From OPU-IVM, six embryos were transferred in three patients 4.25 years after oocyte vitrification, leading to the live birth of a healthy boy. The present case of live birth is among the first cases reported so far and supports the notion that IVM might be a relevant and safe FP option for cancer patients when oocyte preservation is required but ovarian stimulation is contra-indicated.

## 1. Introduction

In the last decade, the incidence rate of different types of cancer in young women has increased considerably, including breast cancer (SEER annual report 2020). The efficacy of early diagnosis is one of the reasons for the increased prevalence of cancer rates accompanied by other technological progresses in anticancer treatment and, consequently, enhanced long-term survival rates of women at reproductive age. Thus, it has become even more important to focus on the late side effects of cancer treatment since pregnancy rates decrease to about 38% post-cancer treatment [1]. Among young women, long-term quality of life is often diminished due to concerns about their future fertility and pregnancy. Advances in reproductive medicine and cryobiology have facilitated an increased interest in fertility preservation (FP) methods. Therefore, international guidelines underlining the significance of counselling every women about the impact of gonadotoxic treatment on future fertility and the possible effects regarding FP are important [2]. In France, the bioethics law imposes such counselling to all health professionals in cases whereby gonadotoxic treatment is required. This imposition benefits patients by smoothing the referral process to a reproductive medicine specialist before the start of oncological treatment. Established FP methods include oocyte and embryo cryopreservation following ovarian stimulation (OS). Ovarian tissue cryopreservation (OTC) is an option for patients who are at pre- or post-puberty and undergoing high-level gonadotoxic and/or urgent treatments [3,4,5,6]. Where applicable, OTC can be used in combination with OS for oocyte cryopreservation in women at a reproductive age [2]. Immature oocytes, retrieved transvaginally from small antral follicles (OPU-IVM) [7,8,9,10,11] or extracted from ovarian tissue ‘ex vivo’ (in vitro-matured ovarian tissue oocytes, i.e., OTO-IVM) [11,12,13], can undergo in vitro maturation (IVM) and be a source of oocytes for cryopreservation. This last methodology resulted in the first births reported from recovered immature oocytes from cancer patients. To date, five births have been reported following OTO-IVM—four derived from vitrified embryos [13,14,15,16] and one from vitrified oocytes [13]. Lately, a few more pregnancies (n = 6) have been also reported following OPU-IVM in cancer patients, derived from vitrified embryos [10,17,18], vitrified zygotes [19], and vitrified oocytes [17,18].

However, in view of the limited number of reported births after IVM in cancer patients, the efficacy of this technology is yet to be established [2]. Data are far from robust and are limited to rates of oocyte recovery and maturation. Reported oocyte maturation rates after 24–48 h culture vary from 23% to 62% from studies involving ‘ex vivo’ extracted oocytes from ovarian tissue [13,17,20,21,22] and between 48 and 67% from immature oocytes collected from transvaginal follicle aspiration [7,8,9,10,23,24,25]. Therefore, an improved understanding of the outcomes of using IVM in specific patients that require oncological treatment is needed.

The good clinical practice of an FP programme requires a multidisciplinary approach that considers the oncologists’ opinion when deciding on the therapeutic strategy for each oncological patient. In France, oncologists contraindicate OS to breast cancer patients in need of neoadjuvant treatment—independent of the hormonal receptivity status of tumours. In the present study, we offer OPU-IVM as an alternative to those patients and report our reproductive results along with the results of OTO-IVM applied in specific indications. We also present the results of the few patients who attempted pregnancy following post-oncological treatment.

## 2. Materials and Methods

### 2.1. Patient Population

This paper presents a retrospective analysis of data from all women diagnosed with cancer who underwent oocyte preservation following OS (OS-FP) or in vitro maturation fertility preservation (IVM-FP) from the start of our FP programme at the IVF center of Inovie Fertilité—Toulouse, France, in January 2014 until December 2019. A total of 122 post-pubertal patients (16–41 years old) were offered oocyte cryopreservation according to their couple status and their personal choices. All cancer types were included. The therapeutic strategy for each patient, including the indication for OS, OTO-IVM, or OPU-IVM was discussed and approved beforehand in our multidisciplinary meeting undertaken with the oncological team from the regional cancer institute (Cancer Institute, IUC-Toulouse) and other IVF centres, with our centre being the reference centre for IVM. The process, timing, and risks associated with the different procedures were discussed among the medical staff and with the patient and her family. Eight of these patients returned to the clinic with a desire to conceive after gonadotoxic treatment, and had their oocytes warmed for ICSI treatment. Written informed consent was obtained from all the patients before each procedure. This study was approved by the local Institutional Review Board.

### 2.2. Ovarian Stimulation (OS)-FP Treatment

Breast cancer patients younger than 42 years old undergoing adjuvant therapy and other cancer types with the potential to achieve stimulation before starting chemotherapy were treated under two types of protocol: the fixed GnRH antagonist protocol—when FSH could start on day 2 of menstrual cycle followed by day 4 or 7 GnRH antagonist administration—or the random GnRH antagonist protocol [3]. It is important to note that none of the patients received aromatase inhibitors as a co-treatment since this medication is not allowed in France. Final oocyte maturation was achieved with 0.2 mg of GnRH agonist when at least two leading follicles reached >18 mm, and oocyte retrieval occurred 36 h later, with oocyte vitrification 2–3 h after retrieval.

## 3. IVM-FP Treatment

### 3.1. OPU-IVM

IVM-FP was indicated in patients younger than 40 years old when chemotherapy could not be delayed or if ovarian stimulation was contraindicated. In fact, most of our OPU-IVM patients were breast cancer patients who had to undergo neoadjuvant therapy for cancer treatment. OPU was performed following the monitoring of patients without any FSH stimulation until, depending on the time available prior to start of oncological treatment, follicles attained diameters between 8 and 12 mm. OPU preferably occurred at the follicular phase of the menstrual cycle or, in cases of emergency FP, in the luteal phase. IVM oocyte retrieval was performed 36–38 h after the subcutaneous or nasal administration of 2 mg GnRH-Agonist. The transvaginal ultrasound-guided retrieval of oocytes was performed using a 19-gauge single lumen needle (K-OPS-7035-RWHET, Cook, Australia) with a reduced aspiration pressure (80 mmHg) under general anaesthesia. Matured oocytes at the day of retrieval were vitrified 3 h post-retrieval.

### 3.2. OTO-IVM

Patients younger than 35 years old underwent unilateral oophorectomy as part of their treatment or because they were identified as patients with a ≥80% chance of being sterile after their oncological treatment and had an indication for OTC. Patients did not receive any type of gonadotropin stimulation prior to surgery. Oophorectomy was performed by laparoscopy in the local operating theatre (adjacent to our IVF laboratory) or in another hospital. In the latter case, ovaries (n = 9) were transported into Leibovitz-15 media (Life Technologies) and supplemented with penicillin/streptomycin mix (Life Technologies) within 1–2 h post-procedure. In all cases, ovaries were transported at 4 °C to minimise the deleterious effect of ischaemic tissue injury and better preserve the ovarian tissue in case reimplantation will be necessary in the future. Upon arrival in the lab, antral follicles were punctured, and the follicle wall was scratched with a scalpel to release the follicle fluid and immature oocytes in the culture dish. Ovarian tissue was trimmed to 1 mm thickness in Leibovitz L-15 medium (Life Technologies, Essonne, France) supplemented with 4 mg/mL human serum albumin (has) (Vitrolife, Goteborg, Sweden), 100 IU/mL penicillin, and 100 µg/mL streptomycin (penicillin/streptomycin mix, Life Technologies), as described elsewhere [16]. Immature oocytes, released from follicles that were ruptured during the OTC process and thus extracted in an ex vivo manner were immediately collected by a second operator under a stereomicroscope at 37 °C. Cumulus oocyte complexes (COC) were washed twice in GMOPS medium (Vitrolife) under oil (Ovoil, Vitrolife) before being placed into culture dish. Naked oocytes were discarded.

**Figure 1:**
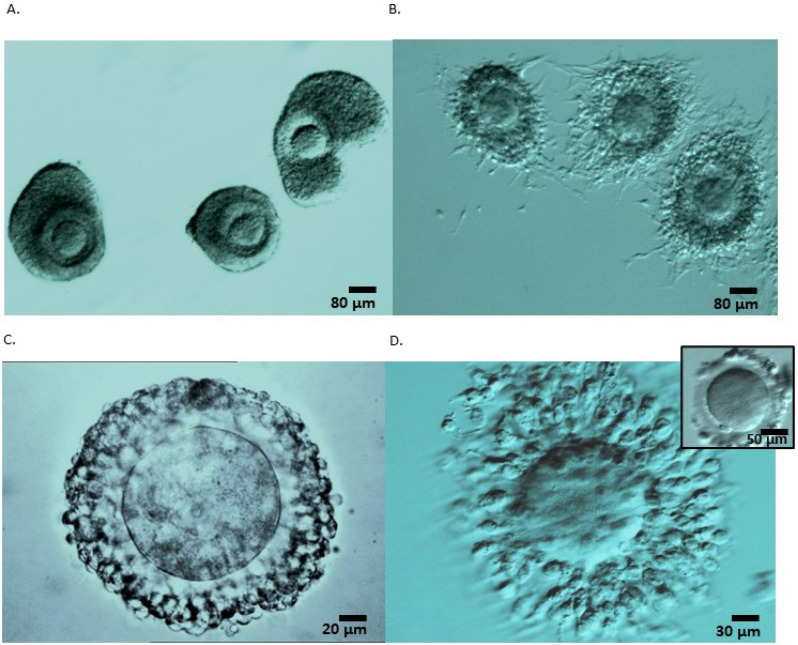
Cumulus enclosed oocytes recovered from ovarian tissue ex vivo (**A**,**B**) and following follicular aspiration (**C**,**D**). (**A**) Cumulus-enclosed GV stage oocytes consisted of retrieved ovarian tissue following dissection and preparation for freezing. These COC constituted of healthy tightly compacted cells surrounding the oocyte despite being recovered from an ovary exposed for 2 h at 4 °C. (**B**) After being in IVM culture for 26 h, the cumulus cells of the matured oocytes expanded and surrounded the oocytes, forming a net of elongated cells partially attached to the dish. (**C**) A compacted cumulus-enclosed GV stage oocyte retrieved following follicle aspiration, and (**D**) after 24 h IVM culture enclosing a mature oocyte; an image of the same oocyte after denudation with the polar body visible at 7 o’clock position (right superior corner). All oocytes were cultured in IVM containing patients’ serum that were not subjected to heat-inactivation.

All immature COC presenting compacted cumulus cells (Figure 1A,C) from both procedures were incubated in IVM medium (IVM System, Medicult, CooperSurgical) supplemented with 75 mIU/mL recFSH (Gonal-F, Merck), 100 mIU/mL recLH (Luveris, Merck), and 10% patients’ serum for a maximum of 48 h in a four-well dish with oil overlay (Ovoil, Vitrolife) in an incubator with the following atmospheric conditions: 6.5% CO_2_ at 37 °C (K-system). In OPU-IVM, some oocytes had expanded cumulus cells in response to the trigger of patients with GnRH agonist. Maturation from those oocytes was observed 3–4 h after retrieval, and as soon as the first polar body extrusion was visualized under the microscope, oocytes were vitrified. If, at this time, oocytes were in the MI stage, they were cultured until mature (the following day). All oocytes underwent vitrification following denudation with or without 40 IU/mL hyaluronidase (SynVitro Hyadase, Cooper Surgical, France). All mature oocytes were cryopreserved using the vitrification method with Vitrification kit media (Kitazato, Fujifilm, Spain), as described by the authors of [26].

### 3.3. Oocyte Warming and Embryo Transfer Procedure

Patients who returned to the centre to attempt pregnancy did so with the approval of their oncologist, and their hormonal replacement therapy (artificial) protocol of endometrium preparation was approved in the FP multidisciplinary meeting.

The warming of oocytes was performed using the kitazato kit method [26]. All vitrified oocytes were warmed, and the surviving oocytes were inseminated using ICSI 2 h after the warming process was completed. Embryos were cultured until day 3 under 5% O_2_, 7% CO_2_ (k-system dry incubator) into G5-series (Vitrolife) and selected for transfer strictly according to their morphological appearance.

Endometrium priming cycle began on the first day of menstruation or after HRT withdrawal caused bleeding in postmenopausal patients. Briefly, the endometrium was primed with oral oestradiol valerate at a dose of 2 mg three times daily. When an endometrial thickness of more than 6 mm was reached, luteal support was provided using intravaginal micronized progesterone tablets (200 mg three times a day) on the evening of oocyte warming, and embryo transfer was scheduled for the fourth day of progesterone administration. Oestrogens and progestins were administered until a pregnancy blood test was performed and continued until 8 weeks of gestation, after which the dose was reduced, and the luteal support was discontinued 1 week later.

## 4. Statistical Analysis

Continuous data are presented as mean ± SD (range) and compared using Kruskal–Wallis rank test, and categorical data are expressed as numbers (percentages) and compared using the Chi-squared test (or Fisher’s exact test where appropriate).

## 5. Results

### 5.1. Patients’ Characteristics

Patients undergoing OPU-IVM (n = 73) included mostly breast cancer patients who had undergone neoadjuvant oncological treatment (80%). In total, 17% of OPU-IVM cases included breast cancer patients undergoing adjuvant oncological treatment who had been referred to our centre too close to the starting date of the chemotherapy treatment or had any other contra-indication for OS. The OTO-IVM (n = 16) cases were identified as patients with a ≥80% chance of being sterile after receiving oncological treatment. The borderline ovarian tumour patients in the OTO-IVM group (n = 6) were patients who had to undergo ovariectomy followed (or not) by chemotherapy in view of suspicious lesions. In most of these cases, OTC was also performed in case future reimplantation is needed or new developments offer the possibility to obtain mature oocytes from her tissue. Most cases of OS included patients with breast cancer receiving an adjuvant treatment (70%). The proportion and numbers of the type of disease in each FP treatment are detailed in Table 1. Side effects were minimal and restricted to one breast cancer patient. This patient was diagnosed with suspected hemoperitoneum occurrence a few days after OPU-IVM. She was discharged after being hospitalised for three days following laparoscopy surgery without the detection of active internal bleeding.

### 5.2. Comparison of Collection Parameters among OTO-IVM, OPU-IVM and OS Procedures

As shown in Table 2, the groups were comparable in terms of FSH level, antral follicle count (AFC), and BMI index. However, given their type of cancer diagnosis, patients in the OTO-IVM group were younger compared to the age of patients undergoing OPU-IVM and OS procedures, who were mostly breast cancer patients.

Out of the 122 patients who underwent treatment for fertility preservation during the study period, 7 did not obtain oocytes after OTO-IVM (n = 2), OPU-IVM (n = 4), and OS (n = 1). In IVM procedures, oocytes were collected during the follicular phase in 62% and 78% of patients and during the luteal phase in 27% and 22% of cycles from OTO-IVM and OPU-IVM, respectively. In 11% of patients whom ovariectomy was performed in another hospital and transported to the laboratory for OTO-IVM, no information was provided regarding the day of the cycle.

### 5.3. Oocytes Collection and Maturation

The number of oocytes collected was higher in OTO-IVM and OS compared to OPU-IVM (*p* < 0.001). All oocytes collected after OTO were at the GV-stage with enclosed compacted cumulus cells (Figure 1), while in OPU, since these patients received GnRH-agonist triggering, 19% of oocytes were already at the MII-stage and 2% were at the MI-stage upon retrieval. In OS patients, 86% of oocytes were MII, 5 oocytes were at the MI stage, and 29 were at the GV stage after denudation. Further in vitro maturation of these collected immature MI/GV oocytes was not performed.

Upon culturing for 24–28 h, the rates of maturation were 16% higher for OPU than for OTO oocytes, and upon culturing for 44–48 h, 82% of GV-stage oocytes ended up maturing to MII from OPU-IVM compared to 70% of OTO-IVM patients (*p* < 0.0001). However, the total number of matured vitrified oocytes was higher for OTO patients (7.6 ± 5.7) compared to those who received OPU (4.6 ± 4.9; *p* < 0.05) and OS (6.8 ± 4.6) (NS) (Table 2). 

There was no difference in maturation rates at the 48 h culture period, when oocytes were extracted “ex vivo” (OTO-IVM) from the unstimulated ovaries during the follicular phase (69%) or luteal phase (68%) in the 89% of patients from whom progesterone level could be obtained to determine the luteal phase. However, from GnRH-agonist-triggered OPU-IVM patients, maturation rates were higher when oocytes were collected during the follicular phase compared to the luteal phase, although these differences were not significant (69% versus 50% at 28 h, and 81% versus 71% at 48 h culture, respectively) (NS). The rate of oocytes that were already matured at MII in OPU-IVM when oocyte retrieval was performed during the follicular phase was 19% versus 13% for luteal phase retrieval (NS). 

Concerning the effect of ovarian transportation on OTO-IVM rates, we observed a lower kinetics of maturation following 1–2 h of exposure at 4 °C compared to immature oocytes from ovaries recovered at our centre and with a shorter exposure time (15–30 min) at 4 °C, which yielded a result of 52% versus 64% at 24 h (NS). After 48 h, a similar maturation was obtained in both conditions (69% versus 66%).

### 5.4. Outcomes of Cryopreserved Oocytes 

A total of seven (6%) out of one-hundred-and-fifteen patients with oocytes cryopreserved in our oncofertility programme were deceased one to two years after cancer diagnosis (four after OTO, two after OPU, and one after OS treatment). Until December 2019, a total of nine (8%) patients returned for oocyte warming (Table 3), and the average of years for these patients’ return following disease-free diagnosis was a mean of 1 year in OTO, 4.25 years in OPU, and 3 years in OS patients. We obtained a relatively low oocyte survival rate (<60%) but a normal proportion of fertilization, which resulted in 35% embryos acquired per total number of oocytes warmed in IVM treatment groups and 50% after OS treatment (NS). Six patients with warmed oocytes underwent the procedure of transferring cleaved embryos, resulting in two clinical pregnancies at 6 weeks, one after OPU-IVM and one after OS. The remaining four patients failed to achieve pregnancy. The OPU-IVM patient was 35 years old, and the OS patient was 38 years old at the time of oocyte vitrification, and both patients were diagnosed with breast cancer prior to FP. These two pregnancies resulted in the delivery of healthy children. The OS patient had a caesarean section at 36.3 weeks of gestation to deliver a boy presenting an Apgar score of 10 and a birth weight and height of 3880 kg and 49 cm, respectively. At one year of age, the boy presented normal developmental growth and cognitive status.

### 5.5. Pregnancy Case from OPU-IVM

In one pregnancy derived from a 35-year-old patient at the time of OPU-IVM treatment from whom eight immature oocytes were retrieved, five oocytes matured 24 h after IVM and were vitrified. The patient returned four years later presenting an ovarian insufficiency with 0.23 ng/mL AMH levels and 21 IU/l FSH after the cessation of her oncological treatment and disease-free diagnosis. Four oocytes survived the warming procedure, and one five-cell embryo was transferred on day 2 of a hormonal replacement therapy (HRT) cycle. After 37.6 weeks of gestation, this resulted in the birth of a healthy boy with an Apgar score 10 and a birth weight and height of 3880 kg and 49 cm, respectively. At one year of age, the developmental growth and cognitive status of the child was reportedly within a normal range.

## 6. Discussion

The presented case of live birth indicates that IVM followed by oocyte vitrification can be applied as a strategy for FP for women in need of oncological treatment. To our knowledge, very few births have been reported in the literature from IVM cases regarding FP in cancer patients. The first live births from OPU-IVM in cancer patients were from vitrified embryos, which were transferred a few years later when patients were in remission [10,17]. The type of oncological condition for each of the patients has not been specified by the authors. The first live birth reported from vitrified IVM oocytes was from a patient diagnosed with invasive breast cancer at the age of 29 [27]. Ovarian stimulation was contra-indicated in view of the cancer type (oestrogen-receptor positive), and the patient decided to undergo IVM only, instead of OTC. Seven immature oocytes were retrieved at OPU, and six oocytes were vitrified 48 h after IVM. The patient returned five years later once deemed cured, became pregnant after the transfer of one cleavage-stage embryo upon endometrial preparation with HRT, and delivered a healthy boy at term. More recently, a live birth following the cryopreservation of zygotes after IVM was reported in a breast cancer patient [19]. A total of two out of four immature oocytes reached maturation, with two zygotes being cryopreserved, the latter of which were thawed 9 years later and cultured until day 3, resulting in a healthy baby at term. The patient was 42 years old by then and had been attempting to conceive naturally without success for 5 years. Subsequently, the single IVM birth described here is thus, to our knowledge, a third reported healthy child from *vitrified oocytes* after IVM in a cancer patient. The patient was diagnosed with breast cancer, and IVM was performed prior to her receiving neoadjuvant oncology treatment at the age of 35. At the time, ovarian stimulation was contra-indicated by the oncologists. Eight immature oocytes were retrieved, with five becoming mature 24 h after IVM. Four years later, when the patient was considered disease-free, she returned with ovarian insufficiency and, following the survival of four oocytes, one five-cell embryo was transferred into an HRT cycle, and the patient delivered a healthy boy. Overall, cases of pregnancy after IVM in cancer patients are recent and data remain limited. The reasons for a lack of data on IVM in cancer patients include the fact that IVM is restricted to certain patient conditions, mainly those that have contra-indication(s) for hormonal stimulation. Another reason for the lack of data on IVM outcomes is because few IVF centres apply IVM routinely and, when considering the risk-benefit balance of the intervention, are often not sufficiently prepared to provide IVM treatment to their FP patients due to a lack of know-how. Additionally, there is the fact that, since the application of IVM together with oocyte vitrification techniques on cancer patients, the return rate of cancer-survivals to recuperate their oocytes is rather low (6–15%) [10,11].

Cryopreservation of the ovarian cortex was made available to cancer patients long before IVM treatment [28,29]. Nowadays, data published by expert centres on ovarian tissue cryopreservation (OTC) and transplantation (OTT) demonstrate that ovarian function after grafting is restored in more than 90% of patients, and approximately half of the >100 reported births have derived from spontaneous conceptions [6,30,31,32]. OTC and OTT are now established and valid options that can be considered in all ages when there is potential for ovarian insufficiency later in a woman’s life, with the most evidence-based utility being in cases of high gonadotoxic treatment [3,4,5]. In this case, OTC can be used as an alternative when oocyte/embryo cryopreservation is not feasible because it has the advantages of being practicable in urgent situations and allows one to forego hormonal treatment. Clinical applications of the procedure to restore ovarian function and fertility were supported by experiments using animal models about thirty years ago, with the first reported success in humans being achieved 15 years later. To increase the chances of future pregnancy, OTC can be combined with other FP strategies, including the following: ovarian stimulation at posteriori to OTC; immature oocyte aspiration at same or collateral ovary followed by OTC [11]. The combination of OTC with oocyte aspiration and cryopreservation seems feasible and effective [12], but the efficacy-related data required to support this conclusion are very limited [2].

In addition, OTC can be combined with the extraction of immature oocytes from the medullar tissue for IVM in a laboratory setting from the surgically removed ovarian biopsies or whole ovaries [11,22]. OTO-IVM is a promising technique for fertility preservation in women who face the risk of losing their fertility potentiality due to cancer treatment, premature ovarian failure, or other medical conditions. Adding OTO-IVM to OTC will not delay cancer treatment and offers a surplus source of oocytes for cryopreservation. For patients with an ovary-related malignancy, experts do not recommend OTT since the putative risk of reintroducing cancer cells seems to outweigh the benefits of the OTT procedure [2]. Alternatively, OTO-IVM is a valuable option to be considered. Five live births have been reported from this methodology, without any reported congenital malformations in the new-born [13,14,15]. In our study, OTO-IVM derived from total oophorectomy resulted in an increase in the number of oocytes retrieved and vitrified when compared to OPU-IVM. It is clear from the literature that the live birth potential of these OTO-IVM oocytes may be significantly lower compared to the live birth potential of mature oocytes retrieved after ovarian stimulation [33]; however, they have, to date, resulted in more live births than oocytes retrieved and vitrified via OPU-IVM.

One of the advantages of IVM, whether by OPU or OTO, is that it can be performed at any stage of the menstrual cycle, which is particularly appropriate when urgent fertility preservation is required prior to oncological treatments [8,9]. Indeed, the retrospective analysis of 192 IVM cycles performed in 164 cancer patients showed no difference between IVM performed during the early follicular, late follicular, and luteal phases in terms of number of oocytes collected, maturation rates, number of cryopreserved oocytes and embryos, and fertilization rates [9]. Furthermore, the prospective analysis of 248 breast cancer patients who underwent fertility preservation prior to neoadjuvant chemotherapy showed similar rates for the retrieval of oocytes, regardless of whether the procedure had been performed in the follicular or luteal phase of the cycle [8]. In our dataset, we did not observe any differences in maturation rates during the follicular phase compared to oocytes extracted ex vivo in the luteal phase (OTO-IVM). However, we could observe higher in vitro maturation rates for oocytes collected from OPU-IVM during the follicular phase compared to the luteal phase (69% vs. 50% at 28 h; 81% vs. 71% at 48 h culture, respectively). Though, given the limited sample size, this difference did not reach statistical significance. It is important to note that, when oocyte retrieval was performed during the follicular phase, the proportion of oocytes that were already matured at MII in OPU-IVM was slightly higher (19% vs. 13% when retrieved at luteal). In view of these preliminary results, when possible, we recommended performing OPU-IVM in the follicular phase rather than in the luteal phase of the cycle in cancer patients.

The success rate of in vitro oocyte maturation for FP can vary depending on several factors, including the age and ovarian reserve of the patient, the protocol used for IVM, and the number and quality of mature oocytes retrieved. Although it has been reported that AFC and serum AMH values >20 follicles and 3.7 ng/mL, respectively, are required to obtain at least 10 IVM oocytes for cryopreservation [25], one must not be discouraged if less oocytes are retrieved. Interestingly, the previously mentioned cases of live births from IVM derived from only a few recovered oocytes. Anyhow, the mean number of oocytes retrieved from IVM is generally less than would be expected after ovarian stimulation, and has been reported to be between five and seventeen in cancer patients [2].

Concerning maturation rates achieved after IVM, reports from the literature describe up to 67% of maturation at 48 h culture when OPU-IVM is performed and ranges from 24 to 57% maturation from oocytes deriving from OTO-IVM [2]. Our overall high rates of maturation of 70% at 48 h of culture in OTO-IVM, and a much higher maturation rate of 82% in OPU-IVM compared to the literature cases might be explained by the type of culture conditions applied. IVM culture involves additives such as hormones, growth factors, and protein sources such as HSA, SSS or patients’ serum. It is common practice to render patients’ serum heat-inactive to avoid concerns over possible contaminants being present in serum and to inactivate the complement binding capacity to avoid cell lysis by antibody binding. This was in fact necessary in routine cell culture, which often involved the use of heterologous serum and serum from animal source. Logically, when using autologous serum, the process of inactivation is obsolete because, firstly, serum is ‘per se’ sterile, and secondly, an immunological reaction will logically not take place. We are the first to report the use of patients’ serum in its innate state, i.e. without heat-inactivation in an IVM culture system. This implies that components such as growth factors, hormones, and other proteins remain intact, favoring the cumulus-cells support to oocyte maturation. However, in the present study, we did not perform a comparison between the two groups (inactivating the serum or not) to effectively demonstrate the differences in maturation rates between the conditions.

It is unclear if our enriched culture system could be the cause for the relatively low survival rates following vitrification. It has been postulated that IVM oocytes are more susceptible to vitrification, and several reports have described the negative effects of vitrification on oocyte survival when IVM is involved [33,34]. However, as data on survival rates after vitrification and warming from oocytes and embryos after IVM in oncological patients are scarce, evaluation on its efficacity is currently unfeasible. The two cases of OS also presented a limited survival rate when compared to our donor oocyte population (56% versus 86%). The low number of patients do not allow us to conclude whether the pathology of the patient influences oocyte quality to vitrification procedure.

It is important to note some other limitations of the present study. Firstly, the study is of a retrospective nature, and data regarding oocyte preservation have been collected throughout a six-year period. This meant that there was a higher chance that any changes related to products other than the IVM medium and culture, or even environmental changes, could have affected the results. Secondly, is the limited sample size, mainly regarding patient return rates, which were fairly low for reasons previously mentioned. Therefore, data on the impact of specific methodologies on oocyte maturation rates presented in this study may not be conclusive. These data are related to the phase of the cycle whereby oocytes are retrieved, whether at the luteal or follicular phase, as well as the influence of the timings of immature oocyte exposure to different temperatures. These aspects should be further analysed with a more robust set of data.

A very important factor to consider is the benefit–risk balance of the OPU-IVM procedure. The methodology of IVM oocyte retrieval is more abrupt than that of conventional OS retrieval. It involves concomitant aspiration and the needle-forced detachment of granulosa cells from the wall of small antral follicles. This increases the risk of blood contamination with respect to follicular fluid and might increase the risk of complications such as internal bleeding post-retrieval. Therefore, IVM requires specific expertise to optimize oocyte retrieval to avoid complications. This might be even more drastic in cancer patients because, if extra eventual intervention or hospitalisation is necessary, their chemotherapy treatment may experience delays. Therefore, it is strongly recommended to perform OPU-IVM only when oocyte cryopreservation is required but ovarian stimulation is not feasible [2], and when patients medical conditions allow.

In summary, with the increase in the life expectancy of patients diagnosed with most types of cancer due to advances in therapeutics and early diagnoses, opening possibilities for different strategies of fertility preservation has become crucial for the improvement of patient quality of life after oncological treatment. IVM technology offers an alternative for FP that otherwise would be not accessible to patients in certain situations. While IVM has shown promising results, it is important to discuss the potential risks and benefits of this technique with a healthcare provider before proceeding. Overall, data on IVM and OTC are still limited with respect to technical consistency, and their efficacy and safety are still to be proven. Prospective studies involving experts in the field could provide additional data to fulfil this aim.

## Figures and Tables

**Table 1:** Proportion of the study population according to the type of cancer and the type of cryopreservation procedure for fertility preservation (FP).

	IVM-FP		
	OTO-IVM	OPU-IVM	OS-FP
Total patients (%)	22 (100%)	73 (100%)	27 (100%)
Breast cancer	1 (4%)	71 (97%)	19 (70%)
Borderline ovarian tumors	6 (27%)	-	5 (19%)
Haematologic malignancy or disorder	4 (18%)	-	2 (7%)
Osteosarcoma	2 (9%)	1 (1.5%)	-
Gastro-intestinal cancer	3 (14%)	-	1 (4%)
Ewing sarcoma	3 (14%)	-	-
Sarcoma other	3 (14%)	1 (1.5%)	-

**Table 2:** Comparison of outcomes from OTO-IVM, OPU-IVM, and OS cryopreservation procedures in cancer patients.

	IVM-FP		
	OTO-IVM	OPU–IVM	OS-FP
Total no. of patients	22	73	27
Total no. of patients with vitrified oocytes	20	69	26
Age at OTC, years ± SD (range)	23.6 ± 6.4 ^a^ (16–32)	30.9 ± 4.2 ^b^ (20–39)	31.8 ± 4.9 ^b^ (19–41)
FSH IU/L, mean ± SD (range)	5.9 ± 2.4 ^a^ (2.1–9.3)	6.4 ± 3.5 ^a^ (2.3–12.0)	7.9 ± 2.3 ^a^ (3.7–12.3)
AMH ng/mL, mean ± SD (range)	5.1 ± 3.7 ^a^ (1.2–15.0)	3.5 ± 3.0 ^ab^ (0.5–13.3)	2.5 ± 1.6 ^b^ (0.4–5.8)
AFC, mean ± SD (range)	17.0 ± 10.5 ^a^ (6–40)	15.6 ± 8.4 ^a^ (7–41)	13.0 ± 5.1 ^a^ (6–23)
BMI (kg/m^2^), mean ± SD (range)	24.1 ± 2.9 ^a^ (21–28)	22.7 ± 5.8 ^a^ (17–38)	23.4 ± 3.7 ^a^ (20–34)
Total no. of oocytes collected (mean ± SD)	223 (11.1 ± 8.1) ^a^	392 (5.4 ± 5.0) ^b^	246 (7.7 ± 5.2) ^a^
Total no. of MI/MII oocytes collected (mean ± SD)	0 (0.0) ^a^	82 (1.2 ± 2.8) ^b^	217 (6.9 ± 4.4) ^c^
Total no. of GV oocytes collected (mean ± SD)	223 (11.1 ± 8.1) ^a^	310 (4.4 ± 3.7) ^b^	29 (1.1 ± 1.8) ^c^
Total no. of MII vitrified oocytes/GV after IVM (mean ± SD)	152 (7.6 ± 5.7) ^a^	254 (3.8 ± 2.8) ^b^	-
% maturation at 24–28 h	57% ^a^	73% ^b^	-
% maturation at 44–48 h	70% ^a^	82% ^b^	-
Total no. of MII vitrified oocytes (mean ± SD)	152 (7.6 ± 5.7) ^ac^	324 (4.6 ± 4.9) ^b^	212 (6.8 ± 4.6) ^c^

^a,b,c^ Different letters denote significant differences among the procedures within the same rows (*p* < 0.01). FP: fertility preservation.

**Table 3:** Outcomes of cryopreserved oocytes in each procedure type of fertility preservation (FP).

	IVM–FP			
Patients Return	OTO–IVM	OPU–IVM	Total IVM-FP	OS-FP
Total no. of patients returned (%)	2 (10%)	4 (6%)	6	3 (12%)
Total no. of oocytes warmed	11	32	43	20
% of oocyte survival	45%	57%	54%	56%
% of fertilization after ICSI	80%	61%	65%	77%
No. of embryos obtained	4	11	15	10
No. of patients with embryo transfer	1	3	4	2
No of embryos transferred	1	6	7	2
Total no. of deliveries	0	1	1	1

## Data Availability

Data is unavailable due to privacy and ethical restrictions.
